# Radiocesium concentrations in wild boars captured within 20 km of the Fukushima Daiichi Nuclear Power Plant

**DOI:** 10.1038/s41598-020-66362-6

**Published:** 2020-06-09

**Authors:** Limeng Cui, Makiko Orita, Yasuyuki Taira, Noboru Takamura

**Affiliations:** 1 Department of Global Health, Medicine and Welfare, Atomic Bomb Disease Institute, Nagasaki University Graduate School of Biomedical Sciences, Nagasaki, Japan; 2 Department of Radiation Protection, Beijing Research Center for Preventive Medicine, Beijing, China

**Keywords:** Environmental sciences, Environmental social sciences

## Abstract

The Fukushima Daiichi Nuclear Power Plant (FDNPP) accident in 2011 released large amounts of artificial radioactive substances into the environment. In this study, we measured the concentration of radiocesium (^134^Cs + ^137^Cs) in 213 muscle samples from wild boars (*Sus scrofa*) captured in Tomioka town, which is located within 20 km of the FDNPP. The results showed that 210 (98.6%) muscle samples still exceeded the regulatory radiocesium limit (100 Bq/kg) for general foods. Radiocesium (^134^Cs + ^137^Cs) levels ranged from 87.1–8,120 Bq/kg fresh mass (FM), with a median concentration of 450 Bq/kg FM. The median committed effective dose was estimated to be 0.070–0.26 μSv/day for females and 0.062–0.30 μSv/day for males. The committed effective dose for one-time ingestion of wild boar meat could be considered extremely low for residents in Tomioka. The relatively high levels of radioactivity found in this study suggest that the high variability of food sources may have led to the large accumulation of radioactive substances. These results suggest that comprehensive long-term monitoring is needed to identify risk factors affecting recovery from a nuclear disaster.

## Introduction

The Fukushima Daiichi Nuclear Power Plant (FDNPP) accident that occurred in 2011 released large amounts of artificial radioactive substances into the environment, particularly cesium-137 (^137^Cs; 8.8 PBq; half-life: 30.2 years), cesium-134 (^134^Cs; 9.0 PBq; half-life: 2.1 years), and iodine-131 (^131^I; 120.0 PBq; half-life: 8 days)^1^. The introduced radionuclides were deposited over a wide area of Fukushima Prefecture and accumulated in local food^2–4^. From April 2012, the Japanese government set the regulatory limit for radiocesium in general foods as 100 Bq/kg^5^.

After the Chernobyl Nuclear Power Plant accident, researchers reported that game animals were contaminated with artificial radionuclides^6–8^. Among all such animals, wild boars showed an especially high radiocesium concentration^9,10^. Gulakov *et al*. measured wild boars captured in a 10–35-km zone from the Chernobyl Nuclear Power Plant in 2008, and found that the average concentration of ^137^Cs in the muscle tissue of wild boars remained as high as 37,000 Bq/kg^11^, even at 22 years after the accident.

Tomioka town (37° 20′43.6″N, 141°0′31″E) is located within 20 km of the FDNPP^12,13^. Immediately after the disaster, almost all residents of Tomioka town were forced to evacuate. The Tomioka town office led infrastructure recovery efforts and decontamination processes to remove radiocesium fallout from the town. On April 2017, the Japanese government lifted the evacuation order for Tomioka town, except for a difficult-to-return zone that comprised almost 15% of the total town area. Although the residential areas, farmland, and forests close to residential areas have been widely decontaminated, it has been reported that the forest area remains contaminated with radionuclides derived from the FDNPP, 8 years since the accident^12^. Highly contaminated wild boars were reported as a considerable issue that led residents to hesitate to return to their hometown. In fact, internal radiation exposure from food remains a matter of concern for the residents of Tomioka town^13^, who wish to know the radioactive levels of wild boar, including the possibility of consuming wild boar in the future. Therefore, the aims of this study were to determine the levels of radiocesium contamination in wild boars found in Tomioka town, Fukushima Prefecture, and, since wild boar is a traditional ingredient in Japanese cuisine, to evaluate the internal radiation exposure risk of consuming wild boar meat.

## Results

### Radioactivity concentration

**Figure 1:**
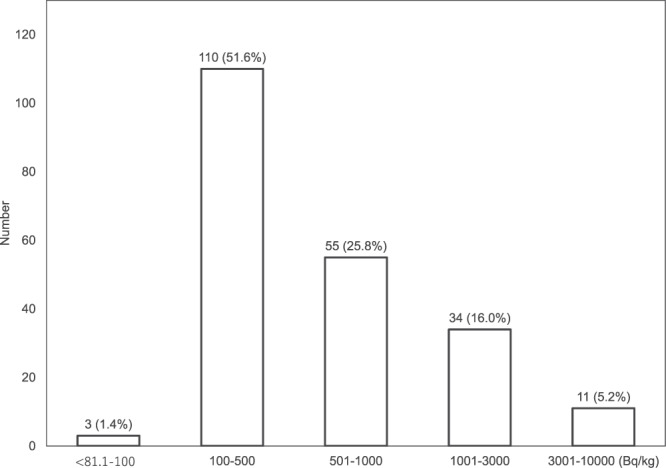
Distribution of radiocesium (^134^Cs + ^137^Cs) concentrations in the muscle tissue of wild boars from January to December 2019.

Among the 213 wild boar (*Sus scrofa*) samples collected, 3 (1.4%), 110 (51.6%), 55 (25.8%), and 45 (21.2%) had radiocesium (^134^Cs + ^137^Cs) levels of <100, 100–500, 501–1,000, and >1,000 Bq/kg fresh mass (FM), respectively (Fig. 1). The minimum and maximum radiocesium concentrations were 87.1 Bq/kg FM and 8,120 Bq/kg FM, respectively, with a median concentration of 450 Bq/kg FM (Table 1). No significant correlation was found between radiocesium concentration and males and females (Mann–Whitney Test, *p* = 0.516) or between radiocesium concentration and the weight of the wild boars (Spearman correlation coefficient, *p* = 0.376). The average ^134^Cs/^137^Cs activity ratios in all samples were 0.08 in January 2019 and 0.06 in December 2019.

**Table 1:** Radionuclide concentrations (Bq/kg FM) in the muscle tissue of wild boars.

N = 213	Median (Minimum–Maximum)
Radiocesium (Bq/kg)	450 (87.1–8,120)
^134^Cs (Bq/kg)	28.6 (n.d.*–509)
^137^Cs (Bq/kg)	420 (81.1–7,610)

*n.d.: could not be determined.

**Figure 2:**
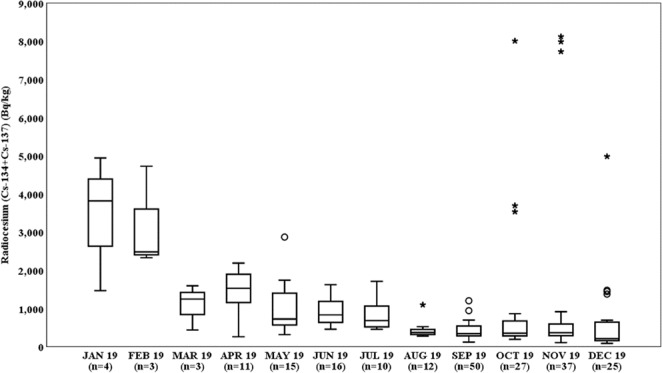
Time-dependency of radiocesium (^134^Cs + ^137^Cs) concentrations in the muscle tissue of wild boars. (Upper error bars: the largest data point excluding any outliers, Lower error bars: the lowest data point excluding any outliers, Open circles: outliers; Asterisks: extreme).

The distribution of radiocesium concentrations in the muscle tissue of wild boars for each month is shown in Fig. 2. Radioactivity concentrations varied significantly with month (Jonckheere–Terpstra test, *p* < 0.05).

### Committed effective dose

Among 213 samples collected that contained radiocesium, the median committed effective dose ranged from 0.070 to 0.26 μSv for females and from 0.062 to 0.30 μSv for males, considering one-time ingestion of wild boar meat as the meat source (Table 2).

**Table 2:** Committed effective doses for one-time ingestion of wild boar meat from Tomioka town (μSv/day).

Age (y)	Female	Male
	Median (Minimum–Maximum)	Median (Minimum–Maximum)
1–6	0.069 (0.012–1.3)	0.072 (0.012–1.4)
7–14	0.18 (0.030–3.7)	0.19 (0.031–3.8)
15–19	0.26 (0.049–4.6)	0.30 (0.058–5.4)
20–29	0.17 (0.032–3.0)	0.18 (0.035–3.3)
30–39	0.15 (0.029–2.7)	0.21 (0.040–3.7)
40–49	0.16 (0.030–2.8)	0.24 (0.046–4.3)
50–59	0.12 (0.023–2.2)	0.18 (0.035–3.3)
60–69	0.070 (0.013–1.2)	0.12 (0.024–2.2)
å 70	NA^*^	0.062 (0.012–1.1)

NA^*^: not available. Median pork consumption was 0 g among women aged >70 years in Japan in 2016.

## Discussion

After the FDNPP accident, Nemoto *et al*. reported that the ^137^Cs concentration of wild boar meat in Fukushima Prefecture from 2011 to 2016 was 900 ± 2,740 Bq/kg FM (mean ± standard deviation [SD]), with a maximum of 40,200 Bq/kg FM^14^. The Fukushima Prefecture government also published data on the radioactivity of wild boars that were captured in the Sousou area of Fukushima (1,737 km^2^), and reported that the highest ^134^Cs + ^137^Cs concentrations from 2011 and 2019 were 5,720 in 2011, 61,000 in 2012, 20,000 in 2013, 30,000 in 2014, 30,000 in 2015, 3,100 in 2016, 14,000 in 2017, 460 in 2018 and 5000 Bq/kg in 2019, respectively^15^. Our results showed a mean ± SD radiocesium concentration of 866 ± 1,270 Bq/kg FM, with a maximum of 8,120 Bq/kg FM. Despite the ^134^Cs/^137^Cs activity ratios in this study agreed with those predicted from physical decay because the average ^134^Cs/^137^Cs activity ratios in all samples were 0.08 in January 2019 and 0.06 in December 2019, our results showed that the wild boar contamination level is still relatively high, even though 8–9 years had passed since the Fukushima accident.

Previous studies in Europe and Japan have reported that about 90% of the diet of wild boars consisted of plants, small animals, insects, and earthworms, based on the season and availability^16–20^, and dietary habits are typically considered an important factor affecting radioactivity levels in wild boars^21,22^. At the same time, the ingestion of soil and deer truffles in winter has also been reported to be one of the causes of radioactive accumulation^8,21^. In 2019, the local government of Tomioka town published the results of an assessment of radiocesium concentrations in locally produced foods by a radioactivity monitoring center. The results showed that the maximum concentration of radiocesium was 99,700 Bq/kg FW in mushrooms, 4,600 Bq/kg FW in edible wild plants, 1,300 Bq/kg FW in chestnuts, 200 Bq/kg FW in persimmons, and 210 Bq/kg FW in bamboo shoots. The radiocesium concentrations in other food types, such as vegetables, potatoes, oranges, and plums, were mostly lower than 100 Bq/kg or not detected^23^. These findings suggest that mushrooms, edible wild plants, and soil with high radioactivity levels were the reasons for the high prevalence of contaminated wild boar in Tomioka town.

If the residents consume the wild boar meat as a meat source once, the median committed effective dose was in the range of 0.062 to 0.30 μSv/day, with a maximum value of 5.4 μSv/day. In Japan, the natural effective dose from food ingestion was estimated to be 99 µSv/y, which was 0.27 µSv/day^24^. Our results indicated that the median committed effective dose from consumption of wild boar meat was similar to or lower than the natural effective dose from food ingestion in Japan. Although, wild boar meat consumption was estimated based on pork consumption in Japan and wild boar meat consumption has been restricted since the FDNPP accident. Thus, the effective dose from the one-time ingestion of wild boar meat could be considered low for residents of Tomioka town.

This study did have some limitations. First, the sample size was small in the first few months of the study. Second, time trends and seasonal variations were still difficult to assess. Actually, seasonal change in radionuclide contamination in wild boar remains controversial^14,25^. Changes in food sources, eating habits, the natural environment, and human behavior may all affect radionuclide concentrations; therefore, continuous measurements are needed to determine how seasonal change affects the concentration of radiocesium in wild boars.

In conclusion, we showed that the wild boar contamination level is still relatively high, even though 8–9 years had passed since the Fukushima accident, but the effective dose from the one-time ingestion of its meat could be considered low for residents of Tomioka town. Long-term monitoring is needed in order to identify a long-term comprehensive risk evaluation such as internal exposure dose for recovery from the Fukushima nuclear disaster.

## Methods

### Sampling information

The Ministry of Agriculture, Forestry and Fisheries of Japan and Tomioka town office have established guidelines regarding the hunting of wild boars. Based on the Act on Special Measures for Prevention of Damage Related to Agriculture, Forestry and Fisheries Caused by Wildlife, Tomioka town office has asked licensed hunters to carry out the capture and processing of wild boars to prevent animal damage to agricultural and forestry products. The wild boars (*Sus scrofa*) were captured using box traps set by the local government and licensed hunters (Fig. 1). The licensed hunters have reported the hunting dates, hunting numbers, and information about the processed boar meat to the Tomioka town office every month. Prior to the study, we obtained approval from the Tomioka town office for use of pieces of legally obtained wild boar meat. In total, 213 pieces of wild boar meat were collected from January to December 2019 (males: 116, females: 97; weight range: 1.1–103 kg).

Samples of fresh wild boar meat (14–108 g) were minced and then enclosed in 100 mL plastic containers made of polypropylene for the radionuclide measurements. All samples were measured fresh and analyzed with a high-purity germanium detector (ORTEC, GMX30–70, ORTEC INTERNATIONAL Inc., Oak Ridge, TN, USA) coupled with a multi-channel analyzer (MCA7600, SEIKO EG&G Co., Ltd., Chiba, Japan). Integration times were 3,600 s for the wild boar samples. The measuring time was set to detect the objective radionuclide, and the gamma-ray peaks used for the measurements were 604.66 keV for ^134^Cs and 661.64 keV for ^137^Cs. Decay corrections were made based on the sampling date, and detector efficiency calibration was performed for different measurement geometries using mixed-activity standard volume sources (Japan Radioisotope Association, Tokyo, Japan). The relative efficiency was 31%, and energy resolution of the spectrometer was 1.85 keV for ^60^Co. The correction factor of the sum-peak effect of ^134^Cs and ^137^Cs were almost 1, respectively. Activity concentrations of radiocesium were automatically adjusted based on the date of collection, and the data were defined as the activity concentrations at the collection date. The counting errors were ±2.9 Bq/kg for ^134^Cs (median) and ±9.5 Bq/kg for ^137^Cs (median), respectively. The ^134^Cs concentrations in 7 samples were lower than the detection limits, which were in the range of 4.1–9.6 Bq/kg. Sample collection, processing, and analysis were executed in accordance with standard methods of radioactivity measurement authorized by the Ministry of Education, Culture, Sports, Science, and Technology, Japan.

### Effective dose

The committed effective doses from the wild boar samples were estimated from the radioactive concentration of the fresh samples using Eq. (1):

1 $${H}_{int}=C\cdot {D}_{int}\cdot {\rm{e}}$$

where C is the activity concentration of the detected artificial radiocesium (Bq/kg FM). Here, D_int_ represents the age-dependent dose conversion coefficients for ^134^Cs (age 1 year, 1.6E-08 Sv/Bq; age 5 years, 1.3E-08 Sv/Bq; age 10 years 1.4E-08 Sv/Bq and age 15–70 years, 1.9E-08 Sv/Bq) and ^137^Cs (age 1 year, 1.2E-08 Sv/Bq; age 5 years, 9.6E-09, age 10 years, 1.0E-08 Sv/Bq; and age 15–70 years, 1.3E-08 Sv/Bq) used in the assessments, which were provided by ICRP Publication 72^26^, and e is quoted from the mean value of daily intake for age and sex. Because wild boar is not a conventional food in Japan, the government and research institutes have not published data on the amount of wild boar consumed. Consequently, wild boar meat consumption was estimated based on the median pork consumption in Japan published by the Ministry of Health, Labour, and Welfare in 2016 (males: 10–49.5 g/day; females: 0–42 g/day)^27^.

### Statistical methods

Data are expressed as medians, minimums, and maximums. Normality was checked using the Kolmogorov–Smirnov test. Because the variables were not normally distributed, non-parametric statistical tests were used. Differences in the concentrations of radiocesium in wild boars at each sampling month were evaluated using the Jonckheere–Terpstra test. Relationships between body weight and the radiocesium concentration in muscle tissue were evaluated using Spearman’s rank correlation analysis. Differences in the concentrations of radiocesium between male and female wild boars were evaluated using the Mann–Whitney U test. P values < 0.05 were considered statistically significant. All statistical analyses were performed using SPSS Statistics 25.0 (IBM Corp., Armonk, NY, USA).

## Data Availability

All relevant data are within the paper.
